# The effect of latency on bone lengthening force and bone mineralization: an investigation using strain gauge mounted on internal distractor device

**DOI:** 10.1186/1475-925X-5-18

**Published:** 2006-03-09

**Authors:** Sekou Singare, Dichen Li, Yaxiong Liu, Zhongying Wu, Jue Wang

**Affiliations:** 1 The key Laboratory of Biomedical Information Engineering of Ministry of Education, and Institute of Biomedical Engineering, Xi'an Jiaotong University, Xi'an, 710049, China; 2 State Key Lab for Manufacturing Systems Engineering, Xi'an Jiaotong University, Xi'an, 710049, China; 3 Oral and Maxillofacial Surgery Department of Bethune International Peace Hospital, Shi Jiazhuang, HeBei Province, 050082, China

## Abstract

**Background:**

The purpose of this study was to investigate the effect of latency on the development of bone lengthening force and bone mineralization during mandible distraction osteogenesis.

**Methods:**

Distraction tensions were investigated at different latency period in 36 rabbits using internal unilateral distractor. Strain gauges were prepared and attached to the distractor to directly assess the level of distraction tension during mandible lengthening. The tensile force environment of the mandible of rabbit during distraction was evaluated through in vivo experiments using two gauges.

The animals were divided into 3 groups each containing 12 rabbits. Latency periods of 0, 4 and 7 days respectively were observed prior to beginning distraction. The distraction protocol consisted of a lengthening rate of 1 mm once daily for 8 days, followed by a consolidation phase of 2 weeks after which the animals were killed. Biopsies specimens were taken from the distracted area at the end of the distraction period. A non-distracted area of the mandible bone served as control. The specimens were analyzed by scanning electron microscopy to assess the ultrastructural pattern, and the bone mineralization.

**Results:**

The resting tension acting on the distraction gap increases through distraction. The 7-day latency groups exhibit higher tension then those of 0-day and 4-days latency groups. Quantitative energy dispersive spectral analysis confirmed that immediate distractions were associated with lower calcium and phosphate atomic weight ratio.

**Conclusion:**

the latency periods could affect the bone lengthening tension and the bone mineralization process.

## Background

Distraction osteogenesis is a surgical process for reconstruction of skeletal deformities. It involves gradual, controlled displacement of surgically created fractures, which results in simultaneous expansion of soft tissue and bone volume. It was first used in limb lengthening by Codivilla [1] in 1905, and later the use of this technique in canine mandible was first reported by Snyder et al [2]. In 1992 McCarthy et al. [3] demonstrated the clinical application of distraction osteogenesis technique in craniofacial skeleton for 4 young patients. To day, distraction osteogenesis has become an accepted method in cranio-maxillofacial surgery to treat severe deformity that could not be adequately corrected with other surgical method.

Mechanical characterization of mandibular distraction osteogenesis is very limited; some analyses of mechanical forces that occur during distraction osteogenesis in leg lengthening have been previously performed [4-13]. These forces have been measured in several experimental studies using a strain gauge mounted on the external distractor device. Recently, Baumgart et al [14] evaluated the traction force during bone transport using strain gauge mounted on the internal distractor device, but the article is published in German. Until now, there are no reports in English literature on the direct measurement of force using a strain gauge mounted on the internal distractor device because of the difficulties in the use of the strain gauge fixed in vivo. Strain gauges must be specially prepared when it has to be used in vivo.

The duration of latency has been investigated in experimental study [15-22] to determine the ideal latency period. However, despite significant clinical experience, little information is known about the influence of the duration of the latency period on the evolution of force during distraction and bone mineralization. In the other hand, the increase of tensile force during distraction osteogenesis will lead to pain or nerve palsy due to overstretching of the soft tissue [23]. Thus, monitoring of this force could prevent the overstretching of soft tissues, supply a method to assess the bone healing [24], and also could provide a means to fix the optimal latency period for distraction osteogenesis.

The present study aims to investigate the effect of latency on the force needed to distract bone using internal distractor, to determine the effect of latency on mineralization of the newly formed bone, and to provide new methods to allow measurement of force developed during distraction using a strain gauge mounted to internal distractor device. A strain gauge is used in vivo to record the force during distraction osteogenesis and to compare the evolution of force at different latency period on the internal fixator during mandible lengthening in rabbit. The mineralization of the newly formed bone at different latency period was also examined by scanning electron microscopy (SEM) and energy dispersive spectral (EDS) analysis.

## Methods

### Rabbit model

36 white male rabbits weighting 2.75 ± 0.25 kg served as the experimental subjects. The animals were divided into three experimental groups such as 0-day latency group (ODL), 4-day latency group (4DL), and 7-day latency group (7DL). Twelve animals were allocated to each of group and underwent unilateral mandibular osteotomies and attachment of an internal distractor device with strain gauge. These experiments were approved by the Committee on Laboratory Animals, School of Stomatology, the Fourth Military Medical University-China.

### Experimental methods

#### Strain gauge surface preparation

For each animal two-miniature strain gauge 120 ohms (BE120-05AA, Micro-Measurements, Zhonghang Electronic Measuring Instruments Co., LTD-China) were used to form one arm of a wheatstone half-bridge circuit. The connecting wires for the electrical circuit are prepared and soldered to the gauges lead and the gauges are attached to the internal distractor. For a suitable gauge protection against humidity caused by blood and organic tissues in vivo, we apply protective coatings over the strain gauge itself and the point where the lead wires are attached. The gauge surface and the wire junction were waterproofed with a small bead of Polyurethane (AZ-709) and then left to dry for the next 15 minutes at ambient temperature, followed by a second layer AZ-709 and then left to dry for the next 24 hours at ambient temperature. The gauges surface was also coated with a layer of medical grade silicone rubber. Then the coated gauge was placed in the vacuum machine for 5 minutes so as to prevent air bubbles being trapped within the insulator. The silicone rubber was then allowed to cure for 24 hour at ambient temperature.

The gauge should always be checked before implantation. However, the insulation using medical grade silicone rubber require 24 hours to be effective; measurements taken shortly after gauge preparation revealed very low insulation resistance. The strain gauges were calibrated each time before the distractor was assembled onto the mandible of the animal. In addition the gauge are sterilized with antiseptics after preparation and used in surgery.

#### Surgery

**Figure 1:**
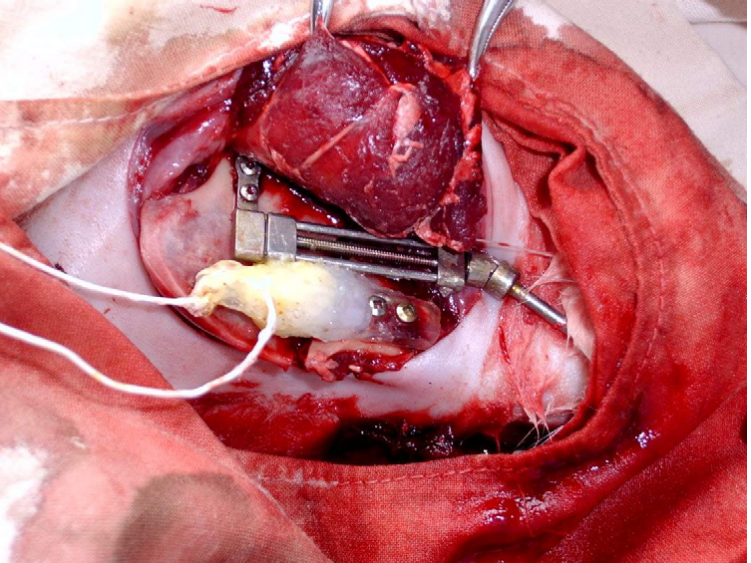
Distraction device and strain gauge fixed along a plane perpendicular to the direction of the osteotomy.

The animals were first allowed to acclimatize to laboratory condition for 4 days and fasted 24 h before surgery. Pentobarbital sodium intramuscularly 1.0 ml/kg was used as a starting dose for anesthesia, followed by a dose of 0.1 ml/kg intramuscularly every 40 minutes to maintain anaesthesia effect. Preoperatively, each animal received 800000 U of intramuscular penicillin sodium 30 minutes before incision. The animal was then positioned in a left lateral position. The right mandible was prepared by shaving and draped after proper sterilization with antiseptics. A longitudinal skin incision was made along the inferior border of the right mandible. The periosteum was incised along the lateral plane of the mandible and was carefully elevated. A bicortical osteotomy was then performed on the buccal aspect with an electric drill (1-mm diameter). About 10 to 15 minutes or even more was required for the bicortical osteotomy. Then the bone was totally separated and the custom made unilateral distraction device with strain gauge was positioned along a plane perpendicular to the direction of the osteotomy with self-tapping screws. The distraction device was then fixed to the mandible with four self-tapping bicortical screws proximal and distal to the osteotomy site (fig. 1). Every effort was made to preserve the inferior alveolar neurovascular bundle. After verifying that the gauges were operational by achieving a balance using Measurements Group Model DH3817 strain gauge conditioners and amplifiers, the periosteum and skin were separately and carefully sutured to cover the osteotomy site and the device was buried under the skin with only the distraction axis passing through the skin. The connecting wires attached to the gauge leads were exited from the wound around the neck. All animals were injected intravenously postoperatively with penicillin sodium antibiotics (400000 U) twice a day for 3 subsequent days.

**Figure 2:**
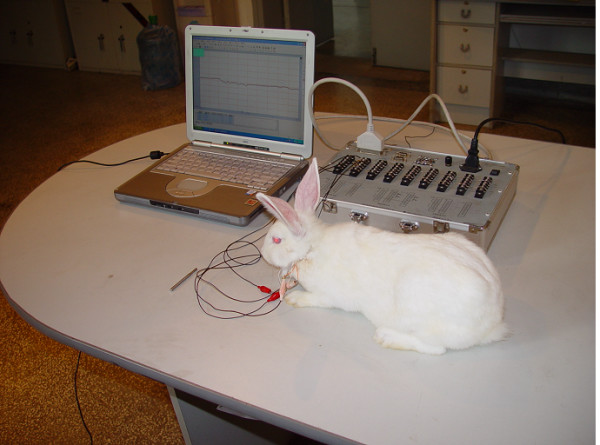
The gauges implanted into the animal are connected to the measurement system. Lengthening procedure. (The rabbit is keep calm and peaceful on its legs during the lengthening procedure).

After 0, 4 and 7 days latency, distraction was performed at a rate of 1 mm once a day for 8 consecutive days. All rabbit underwent strain gauge measurement during bone elongation phase (fig. 2). The animals were sacrificed two weeks after the completion of distraction for SEM examination. A biopsy from non-distracted area served as control.

### Statistical analysis

The data are presented as mean ± standard deviation (SD). The data were analyzed using SPSS software (SPSS Inc, Chicago, IL; 10.0 edition for Windows). Two-way analysis of variance (ANOVA) was used to test for latency or lengthening distance effects on distraction tension. To determine if the bone mineralization at different latency groups were significantly different from each other, one-way analysis of variance (ANOVA) was performed. If this indicated a significant result, Student-Newman-Keuls method for multiple comparisons among the experimental groups was applied to examine whether or not the bone mineralization in each pair of different experimental group and also experimental group versus control group were significantly different at an overall 0.05 type I error level.

## Results

### In vivo force measurement

A total of 36 rabbit were operated and a total achieved length by the end of distraction was for all rabbits 8 mm. During our experimental study local infection at the exit site of the connecting wire was a minor problem in 3 rabbits. This infection was treated with surgical drainage and antibiotics.

The distraction tensions were measured during the course of distraction and continuously for five minutes after distraction. During the distraction process, a sharp increased of distraction tension was noted, after 1 mm increment the distraction tension slowly decreased to a plateau. The importance of measuring the distraction tension 5-minutes after lengthening is that at this time the forces start to stabilize. So the force measured 5 minutes after distraction represent the steady state force.

**Figure 3:**
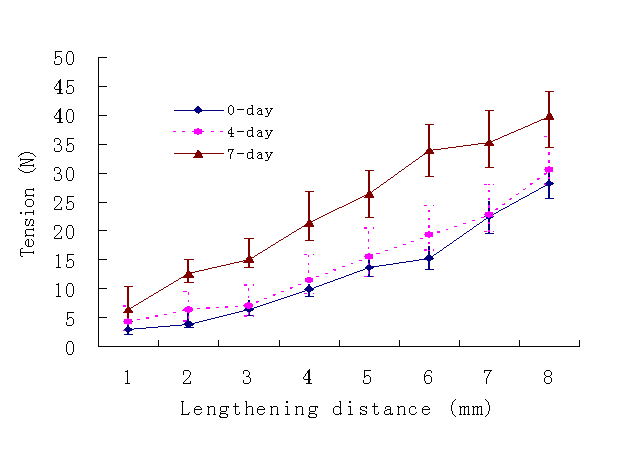
In vivo Post-distraction tension after 5 minute measured during the 8-day distraction period at different latency period.

Values for post-distraction tension averaged across all mandibles are shown in table 1. The tension in all rabbit increased with postoperative time with an average resting tension ranging between 28.23 ± 02.64 N and 39.86 ± 04.30 N at the completion of distraction (fig. 3). The means and standard deviation (± SD) for tension generated during lengthening phase in 0, 4 and 7 days latency with 1 mm once a day was 12.88 N ± 09.00, 14.65 N ± 09.12 and 19.39 N ± 10.64 respectively. There was an increase of tension related to latency period, the tension of 7 day latency was higher than those of 4 day latency, and the tension of 4 day latency was slightly higher than those of 0 day latency. The two-way ANOVA analysis demonstrated that there was a strong effect of both factor such as latencies and lengthening distances on the distraction tension (P < 0.001). The lengthening distance and the latency period had a significant effect on the increase of tension during the lengthening process. The two-way ANOVA analysis further indicated that the latency period and the lengthening distance did not affect each other significantly (P = 0.58).

**Table 1:** Post-distraction tension measured 5 minutes after distraction

Distance (mm)	0DL (N, SD)	4DL (N, SD)	7DL (N, SD)
1	3.02 (0.90)	4.28 (1.70)	6.43 (2.10)
2	3.89 (0.50)	6.38 (2.10)	12.61 (2.50)
3	6.43 (1.01)	7.06 (3.54)	15.16 (2.01)
4	9.93 (1.30)	11.47 (4.20)	21.40 (3.15)
5	13.70 (1.50)	15.53 (4.41)	26.46 (4.70)
6	15.30 (2.25)	19.26 (4.07)	33.90 (4.15)
7	22.56 (3.11)	22.71 (4.34)	35.29 (4.90)
8	28.23 (2.64)	30.51 (3.41)	39.86 (4.30)

ODL = 0-day latency group, 4DL = 4-day latency group, 7DL = 7-day latency group

### Scanning electron microscopy

**Figure 4:**
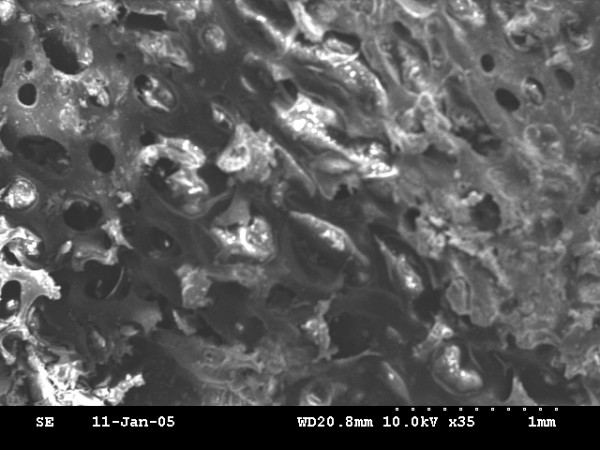
0-day latency: SEM micrograph view of "honey-comb" like structure of immature new formed bone trabeculae with abundant osteoblasts embedded into lacuna × 35.

**Figure 5:**
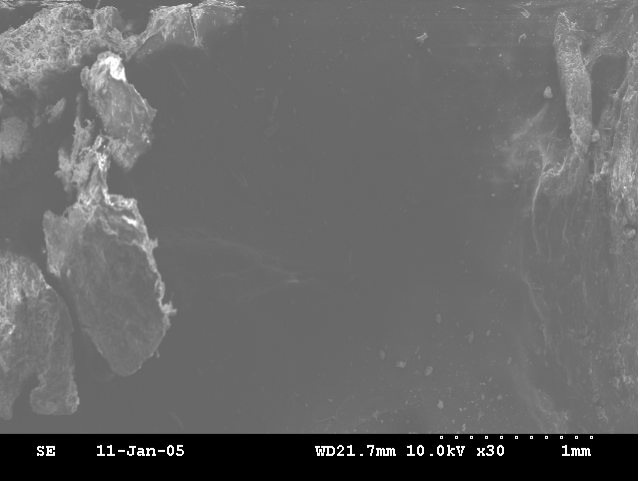
The most central zone of the 0-day latency group, observation of low density of SEM micrograph demonstrated fibrous tissue with some osteoid were scattering × 30.

**Figure 6:**
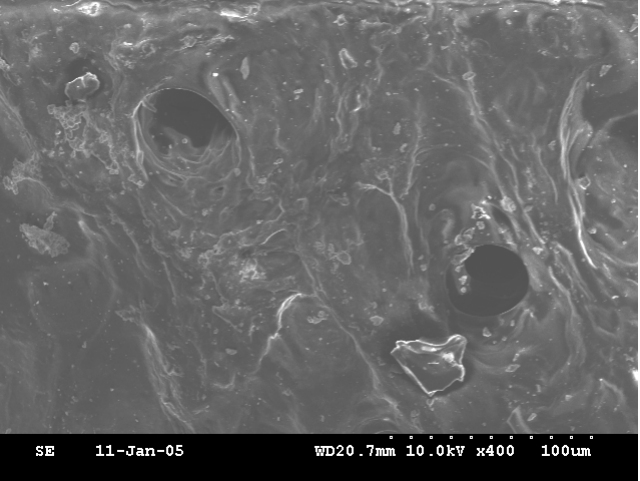
4-day latency: SEM micrograph view of immature new formed bone with osteoblasts embedded into lacuna × 400.

**Figure 7:**
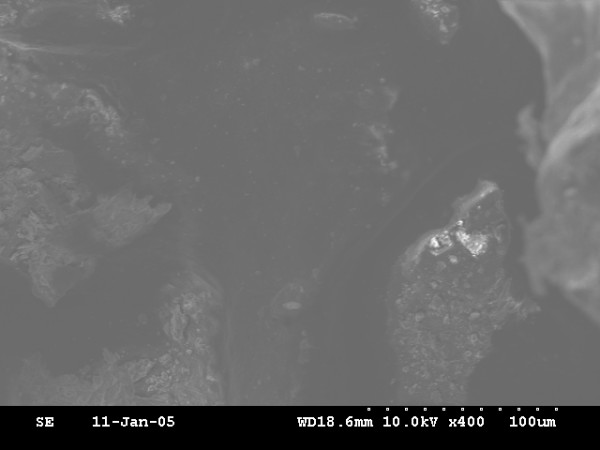
The most central zone of 4-day latency group, observation of low density of SEM micrograph demonstrated fibrous tissue with some osteoid were scattering × 400.

**Figure 8:**
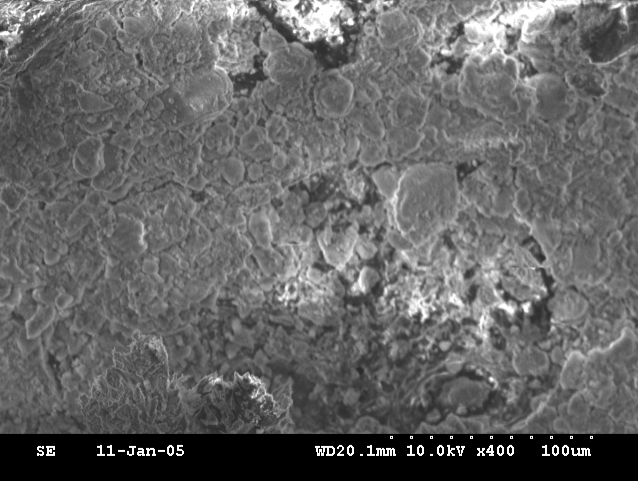
SEM micrograph view of 7-day latency: The gap was totally filled with dense new bone trabecula.

Newly bone is formed in all animals. On the basis of ultrastructural analysis, the newly formed bone of the 0-day and 4-day latency period group showed honey-comb like structure of immature bone trabeculae with abundant osteoblasts in bone lacuna (fig. 4, fig. 6), but the 0-day distraction group showed more cavity in bone microstructure than that of the 4-day latency group. The most central zone, displayed a small area of fibrous tissue with some osteoid (fig. 5 and fig. 7). However, the 7-day latency period groups exhibit mature new bone trabecula structure, which totally fill the distraction gap (fig. 8).

The calcium and phosphorus was used to assess the composition of the mineralized regions of the mandible. Table 2 shows the Ca/P weight ratio of the calcium phosphate atomic weight calculated from the wavelength energy dispersive spectral (EDS) analysis. The ratio of Ca to P, the principal mineral components, is lower in specimens distracted at 0-day latency (1.43) and 4-day latency (1.45). There are no differences in bone mineralization between the 0-day and 4-day latency (P = 0.15). The 0-day and 4-day had a significant reduction in bone mineralization compare to the non-distracted side (P < 0.05). However, in specimens distracted at 7-days latency, increase mineralization was found (1.62), and remains inferior to the control area (1.97). There was statistical difference in bone mineralization after 7-day latency compare to the 0-day and 4-day latency (P < 0.05).

**Table 2:** Atomic weight of calcium and phosphorus expressed as % measured in the distracted bone versus non-distracted bone

	Latency			Non distracted bone
	0-day	4-day	7-day	
Ca	58.85 ± 1.03	59.18 ± 2.02	61.83 ± 1.08	66.32 ± 3.05
P	41.15 ± 0.02	40.82 ± 1.01	38.17 ± 2.04	33.67 ± 2.08
Ca/P	1.43	1.45	1.62	1.97

## Discussion

The aim of the present study was to determine the magnitude of forces engendered in mesenchymal gap tissue during distraction at different latency period. The mandible distraction tension is influenced by many factors such as: the stiffness of the callus, the muscle and soft tissue that surround the osteotomy site and the chewing. In this study the rabbits were keep calm and peaceful on its legs during tension measurement. In some case when the rabbit is not peaceful, an anesthetic is recurred to allow accurate measurement. In that cases the influences of chewing were tolerated, therefore, the observed distraction tension is shared between regenerate and soft tissues themselves and the fixator device. Immediately after separating the two bones fragment, the fixator device experiences compressive deformation, which is nearly equal in magnitude but opposite in direction to the forces transmitted through both regenerate and soft tissues.

The tension during distraction has been measured in several experimental studies by various authors [4-13,24]. But there are no data on the evolution of tension, microstructure of bone formation and bone mineralization due to latency periods. In this study, the effect of latency is investigated by using the strain gauges mounted in the internal distractor device and the regenerated bone is evaluated by ultrastructural and mineral analysis.

The result shows that the latency period had a strong effect on the increase of distraction tension. In the immediate distraction group (0 day latency) the postdistraction tension registered was 28.71 N, whereas in the 4-day delay distraction group, the tensile was 30.51 N. In the 7-day delay distraction group, the tensile was 39.86 N. This result is in agreement the data reported by White and Kenwright [12] in adult male New Zealand white rabbit tibia model in which the bone was lengthened for 20 day for a total length of 10 mm at 0.5 mm once daily. The tension obtained at the end of distraction in the immediate distraction group was about 30 N, and in the 7-day delay distraction group was more than 50 N.

Our study has shown that the latency period has an impact on bone mineralization in the distraction gap. The 0-day and 4-day latency period were followed by slower bone mineralization. In contrast, a delay of 7-days before distraction resulted in a higher mineralization. Experimental studies have shown that a delayed distraction, compared with immediate, could improve the quality of the callus with quicker, denser, and more homogeneous bone formation [15,20]. White and Kenwright [22] found that an experimental osteotomies subjected to immediate distraction result in the production of a small volume of callus with deficient vascularity whereas delayed distraction has been shown to promote increased callous volume and capillary ingrowth. As one would expect, there was no small volume of callus found in the current study as determined by gross macroscopic in the immediate distraction group. Anatomic difference between the leg and mandible also could contribute to the difference seen in the callus volume. In addition, the vascular soft tissue envelope of the craniofacial skeleton may be responsible for successful distraction when using immediate distraction. The current study data are not similar to data reported by Paccino [19] who found that a latency period of 7 days had a subjective decrease in the quality of the regenerate bone when compared with a latency period of 5 days. This discrepancy may be related to differences in other factors that influence the osteogenic response during distraction, such as the type of animals, surgical technique, the mechanical condition of fixation and also the type of the osteotomie.

In the other hand, this study found no differences in bone mineralization in a moderate range of latency periods (0-day and 4-day latency). This is consistent with the findings of Troulis et al [25] and Glowacki et al [16] through distraction in porcine mandible after 0- or 4-day latency with 14 days fixation found equivalent healing. However, Tavakoli et al [17] in sheep mandible in which distraction were started after 0-, 4- and 7-day latency with a rate of 0.5 mm twice daily for 20 day follows by 20 days of neutral fixation found equivalent bone formation. Aronson and Shen [18] in canine model perform distraction at 0, 7, 14, and 21-days latency period with a rate of 1 mm per day for four weeks reported that latency was not required in distraction osteogenesis of canine long bones. The lengthening distance, the rate of distraction and the consolidation period could be the factor for equivalent healing.

There are mutual dependencies between the magnitude of distraction tension and latency period. The 0-day and 4-day latency period causes decreased distraction tension with less bone mineralization, but with time the regenerate mineralization will increase and reach the normal value during the maturation process. However the 7-day latency period can cause increased distraction tension with more bone mineralization. Therefore, the high tension (7-day latency) suggests good bone mineralization and should be considered preferably. However, Galardi et al [26] found that serious complication such as tissue damage, pain or nerve palsy may occur as the tensile force increase with lengthening. If most of the distraction tension is absorbed by the soft tissues, the increased distraction tension may affect soft tissue structure such as inadequate muscular grow or soft tissue damage and thereby decreased distraction tension (i.e. 0-day or 4-day latency) are desired to prevent the soft tissue overstretching.

From a clinical point of view, these experimental data show that decreasing or eliminating the latency period may slow down bone mineralization, but not cause tissue damage due to the lower level of distraction tension, nor hinder new bone formation. However, long consolidation time may then be required in order to achieve required bony filling of the gap.
